# Design of Piezoelectric Ultrasonic Composite Vibration System for Precision Grinding

**DOI:** 10.3390/mi16040408

**Published:** 2025-03-30

**Authors:** Weiqing Huang, Kaijie Huang, Qunyou Zhong, Jialun Wu, Dawei An

**Affiliations:** School of Mechanical and Electrical Engineering, Guangzhou University, Guangzhou 510006, China; meehuangweiqing@gzhu.edu.cn (W.H.); 2112207005@e.gzhu.edu.cn (K.H.); qunyz@e.gzhu.edu.cn (Q.Z.); 2112307088@e.gzhu.edu.cn (J.W.)

**Keywords:** ultrasonic grinding, longitudinal–torsional vibration, sapphire wafer grinding, resonance frequency, surface morphology

## Abstract

Due to the high hardness and brittleness of sapphire, traditional machining methods are prone to surface scratches and microcracks. As an advanced processing technique, ultrasonic machining can reduce damage to hard–brittle materials and improve surface quality. In this study, an integrated ultrasonic longitudinal–torsional vibration system consisting of both a horn and a tool was designed. The resonant frequency and output amplitude of the horn were simulated and tested. The results indicated that the resonant frequency was 19.857 kHz, the longitudinal amplitude at the tool end was 4.2 µm, and the torsional amplitude was 1.8 µm. Experiments were then carried out to investigate the effects of various machining parameters on the reduction of sapphire surface roughness (Ra) and material removal rate (MRR). A comparative experiment was then conducted to evaluate the effects of ultrasonic longitudinal and longitudinal–torsional vibration on sapphire grinding. The ultrasonic longitudinal–torsional grinding experiments showed that the surface roughness of the sapphire workpiece was reduced from 960.6 nm to 82.6 nm, and the surface flatness was improved to 84.3 nm. Compared with longitudinal ultrasonic vibration, longitudinal torsional grinding reduced the surface roughness of sapphire workpieces by 48% and increased the surface flatness by 88.3%. The results of this study provide specific guidance for the longitudinal–torsional composite ultrasonic machining of hard–brittle materials.

## 1. Introduction

As the core component of the ultrasonic vibration machining system, the ultrasonic transducer and horn’s geometric design and material selection will directly affect the transmission efficiency and processing effect of the vibration energy [1,2]. Studies have shown that by optimizing the design of the horn, the stability of energy transfer can be significantly improved, and the effective stress of the horn can be reduced, thereby more effectively improving the surface processing quality of hard–brittle materials [3,4,5]. Satpute et al. designed a two-dimensional resonant ultrasonic transducer capable of generating elliptical motion. Compared to conventional slot milling processes, this novel two-dimensional ultrasonic–assisted vibration machining system results in a 162% Reduction in the surface roughness of titanium alloy workpieces [6]. Baraya et al. found that the machining accuracy of the slotting process is better than that of traditional turning under the ultrasonic vibration device with a frequency of 34.7 kHz and amplitude of 10 µm [7]. Choi et al. designed an ultrasonic tool horn with a vibration frequency of 20 kHz for ultrasonic assisted grinding. They found that the grinding force of ultrasonic grinding was 26% lower than that of conventional grinding [8]. There are three modes of two-dimensional ultrasonic vibration, which are longitudinal–torsional, longitudinal–bending, and torsional–bending vibration. Due to the vibration in different directions, the free abrasive flow and chip removal in ultrasonic machining are improved [9,10]. Ye et al. conducted a two-dimensional ultrasonic longitudinal–torsional grinding test on SiC materials, the grinding force of ultrasonic longitudinal–torsional grinding was reduced by 62.26%, and the surface roughness was increased by 22.78% compared with conventional grinding [11]. An et al. designed a longitudinal–torsional composite piezoelectric ultrasonic transducer for high–precision machining. Experimental results indicated that under a preload torque of 70 Nm, the resonant frequency was 21.695 kHz, and the amplitude met the requirements for ultrasonic machining [12]. Wu et al. proposed a transducer that can realize the joint output of longitudinal and torsional vibrations of shared vibration nodes. At a peak–to–peak voltage of 500 V, the maximum longitudinal displacement reaches 6 µm and the torsional displacement reaches 11 mrad. The results of ultrasonic–assisted milling show that the transducer can significantly reduce the cutting force [13].

Sapphire is a single–crystal form of aluminum oxide with a hexagonal close–packed crystalline structure. Its Mohs hardness is 9, making it exceptionally hard and wear–resistant. Unlike polycrystalline corundum, sapphire exhibits anisotropic mechanical properties due to its monocrystalline nature, making it highly resistant to plastic deformation but prone to brittle fracture along specific crystallographic planes. Due to its optical transparency and thermal stability, these features have resulted in extensive usage in semiconductors, optical equipment, and applications in extreme environments [14,15]. However, owing to their hard–brittle nature, traditional processing methods, such as grinding and milling, lead to problems such as cracks, scratches, and tool wear during processing, which affect processing efficiency and quality. To address the above issues, ultrasonic vibration has been introduced into traditional machining methods, including water jet and abrasive flow machining [16,17]. They found that with the addition of ultrasonic vibration, the material removal rate of the workpiece is significantly increased, and surface roughness is improved. This is attributed to ultrasonic vibration, which can effectively enhance the contact between the abrasive and the workpiece surface, improving the surface quality. Ultrasonic vibration grinding has a critical application in material processing. The high–frequency vibration of the ultrasonic wave during the machining process can provide impact kinetic energy for the abrasive particles. Micro–cutting and plowing occur when abrasive particles impact the workpiece surface, thereby achieving material removal from the workpiece [18,19]. Compared with traditional grinding, ultrasonic grinding machining has a higher machining accuracy, less machining heat, and reduced workpiece deformation and residual stress; therefore, the study of ultrasonic machining has high practical significance and application prospects [20,21,22,23]. An et al. proposed a piezoelectric ultrasonic local resonance ultraprecision grinding technology. Research shows that ultrasonic local resonance grinding can reduce the surface roughness of sapphire by 90.79%, increase the surface flatness by 81.58%, and increase the material removal rate by 31.35% [24]. Xu et al. carried out longitudinal–torsional composite ultrasonic vibration end grinding on sapphire. They found that the surface roughness of the workpiece can reach a minimum of 0.522 µm. Compared with the traditional grinding method, the roughness is reduced by 20.98% [25]. Zarepour et al. proposed a model for predicting material removal modes in micro–ultrasonic machining [26]. Ichida’s experiments show that there are three primary forms of material removal in noncontact ultrasonic grinding machining [27]. Pandey’s dynamic–impact model predicts cross–section profiles of glass vias created by the ultrasonic micromachining, with experimental validation showing the importance of abrasive impact velocity, ultrasonic power ratings, and tool–workpiece gap in effective glass machining [28]. Therefore, ultrasonic machining offers significant advantages in improving processing efficiency, reducing surface roughness, and optimizing machining precision. Further exploration of the effects of different ultrasonic vibration modes and process parameters on machining outcomes is of critical theoretical significance and practical application value.

Current research on ultrasonic vibration machining is focused on the optimization of horn design and frequency coupling to balance energy delivery and vibration stability, which is considered crucial for prolonged ultrasonic machining. While ultrasonic vibration machining has demonstrated potential in enhancing material removal and surface quality, current research predominantly focuses on single–axis vibration modes, limiting the exploration of synergistic effects from multi–dimensional vibration coupling. Additionally, existing studies on process parameter optimization, under multi–axis vibration modes remain fragmented, particularly for ultrahard materials like sapphire.

Therefore, this paper introduces the two-dimensional ultrasonic longitudinal–torsional grinding method into sapphire processing, designs a two-dimensional ultrasonic longitudinal–torsional horn structure, and conducts simulation analysis on the horn’s resonant frequency and output amplitude. Subsequently, experiments on the ultrasonic longitudinal–torsional processing of sapphire were carried out. The experimental results indicate that this method can significantly improve the material removal rate and surface quality, providing a new technical approach for processing hard–brittle materials such as sapphire.

The structure of this paper is as follows: Section 2 presents the materials and methods, including the design of the ultrasonic vibration grinding system, finite element analysis, performance testing, and experimental setup; Section 3 discusses the results and analysis, focusing on the reduction of surface roughness and material removal rate, as well as the surface morphology of sapphire; and Section 4 concludes the paper with key findings.

## 2. Materials and Methods

### 2.1. Design of Ultrasonic Vibration Grinding System

**Figure 1:**
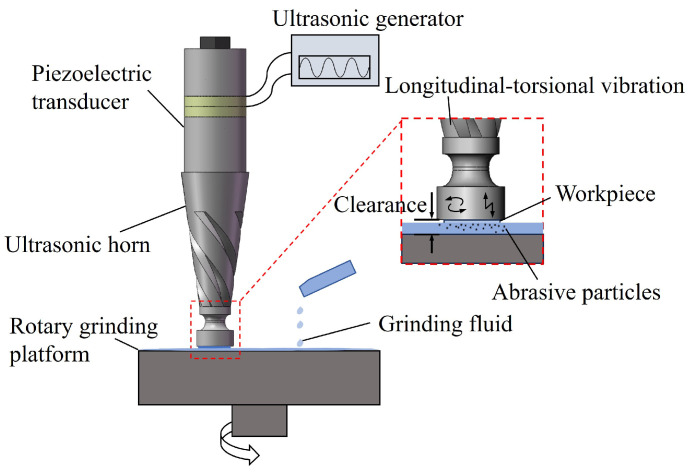
Principle diagram of the ultrasonic grinding system.

The ultrasonic vibration grinding system is shown in Figure 1, composed of a piezoelectric transducer, ultrasonic horn, and rotary grinding platform. The piezoelectric transducer converts electrical energy into mechanical vibration energy, and the ultrasonic horn amplifies the mechanical vibration, with the amplitude reaching its maximum value at the tool face. The ultrasonic vibration excites the abrasive in the grinding fluid to impact the surface of the workpiece, thereby achieving precision grinding.

**Figure 2:**
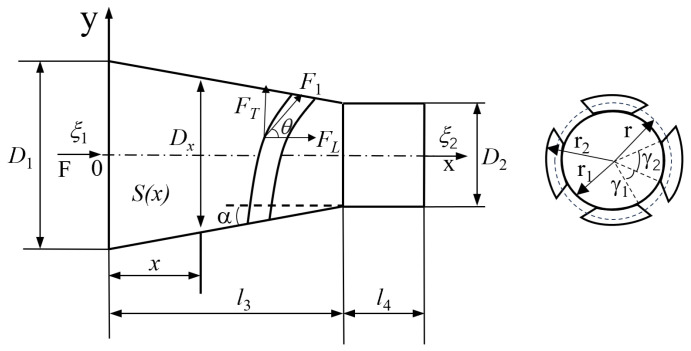
Conical–cylindrical composite horn structure diagram.

The design method for different types of horns varies based on the variation law of cross–sectional area. For a cone–cylinder composite horn, the corresponding vibration equation can be obtained using the shape function of the horn at all levels, and the frequency equation can then be derived based on the boundary conditions. A structural diagram of the cone–cylinder composite horn is shown in Figure 2.

The wave equation of any variable cross section can be obtained as follows [29]:

(1) $$\frac{\partial^{2}\xi }{\partial x^{2}}+\frac{1}{S}\cdot \frac{\partial S}{\partial x}\cdot \frac{\partial \xi }{\partial x}+k^{2}\xi =0$$

where *S* is the cross–section at any point of the composite horn, ξ(x) is the displacement function of the particle, *k* is the circular wave number, k=ω/c=2πf/c, and *c* is the propagation speed of the ultrasonic waves in the material.

In the conical section of the composite horn, $\xi_{1}\left(x\right)$ is as follows:

(2) $$\xi_{1}\left(x\right)=\frac{A_{1}\cos\left(kx\right)+B_{1}\sin\left(kx\right)}{x-1/a},0\leq x\leq l_{3}$$

(3) $$\begin{array}{c} \frac{\partial \xi_{1}}{\partial x}=\frac{-A_{1}k\sin\left(kx\right)+B_{1}k\cos\left(kx\right)}{x-1/a}\\ -\frac{A_{1}\cos\left(kx\right)+B_{1}\sin\left(kx\right)}{{\left(x-1/a\right)}^{2}} \end{array}$$

where *a* is the taper coefficient, $a=\left(D_{1}-D_{2}\right)\left(D_{1}l_{3}\right)$, and $A_{1}$ and $B_{1}$ are undetermined coefficients.

In the cylindrical section of the composite horn, $\xi_{2}\left(x\right)$ is as fiollows:

(4) $$\xi_{2}\left(x\right)=A_{1}\cos\left(kx\right)+B_{1}\sin\left(kx\right),l_{3}\leq x\leq l_{3}+l_{4}$$

(5) $$\frac{\partial \xi_{2}}{\partial x}=-A_{1}k\sin\left(kx\right)+B_{1}k\cos\left(kx\right)\left(l_{3}\leq x\leq l_{3}+l_{4}\right)$$

According to the continuity of the boundary conditions of displacement and force, the vibration frequency equation can be obtained as:

(6) $$\tan\left(kl_{4}\right)=\frac{D_{1}}{D_{2}}\cdot \frac{a}{k}-\tan\left[kl_{3}+\arctan\left(\frac{a}{k}\right)\right]$$

From the above structural design theory, it can be deduced that $l_{3}$ is 85 mm and $l_{4}$ is 10 mm.

The longitudinal inertia force *F* generated by the longitudinal wave of the transducer, when passing through the helical groove of the amplitude transformer, is divided into a longitudinal component $F_{L}$ and a torsional shear component $F_{T}$. As shown in Figure 2 when the helical angle of the groove is θ, the force components can be expressed as:

(7) $$\;F_{L}=F_{1}\cos\left(\theta \right),F_{T}=F_{1}\sin\left(\theta \right)$$

Due to the presence of shear force, the torque *M* generated by the horn can be expressed as [30]:

(8) $$\begin{array}{cc} M & =\int_{r_{1}}^{r_{2}}r\cdot \frac{360F_{1}\sin\left(\theta \right)}{\left[\left(\gamma_{1}\pi r_{1}^{2}\right)+\left(\gamma_{2}\pi r^{2}\right)\right]}\cdot \frac{\gamma_{2}\pi r}{180}\;dr \end{array}$$

Solving the integral in Equation (8), the following can be obtained:

(9) $$\begin{array}{c} M=2F_{1}\sin\theta \left[\left(r_{2}-r_{1}\right)-r_{1}\sqrt{\frac{\gamma_{1}}{\gamma_{2}}}\arctan\left(\frac{r_{2}}{r_{1}}\cdot \sqrt{\frac{\gamma_{2}}{\gamma_{1}}}\right)-\arctan\sqrt{\frac{\gamma_{2}}{\gamma_{1}}}\right] \end{array}$$

where $r_{1}=D_{2}/2$, $r_{2}$ is the radius of the cross–section corresponding to the maximum value of $D_{x}$, $\gamma_{1}$ is the central angle corresponding to the two cut helical grooves in the section, and $\gamma_{2}$ is the central angle corresponding to the uncut portion of the helical groove in the section. From Figure 2, the cone angle α of the horn’s conical section can be expressed as:

(10) $$\tan\alpha =\frac{\Delta y}{\Delta x}=\frac{D_{1}-D_{2}}{2l_{3}}=\frac{D_{x}-D_{2}}{2(l_{3}-x)}$$

Combined with the above derivation, the integrated resonance frequency of the horn tool was designed to be approximately 20 kHz, and the material used was carbon steel. The diameter of the large end of horn $D_{1}$ was 55 mm, and the diameter of the small section $D_{2}$ was 30 mm. There were four spiral grooves, the pitch was 120 mm, the number of turns was 0.5, and the depth of the spiral groove was 6 mm.

### 2.2. Finite Element Analysis of Vibration System

The finite element software COMSOL Multiphysics 6.1 was used to simulate and analyze the resonance frequency of the ultrasonic longitudinal–torsional vibration system. The material of each ultrasonic longitudinal–torsional vibration system component is listed in Table 1. The horn is made of medium carbon steel C45. It underwent quenching and tempering to achieve a hardness of approximately 45–50 HRC, ensuring improved wear resistance, fatigue strength, and structural stability under ultrasonic vibration.

**Figure 3:**
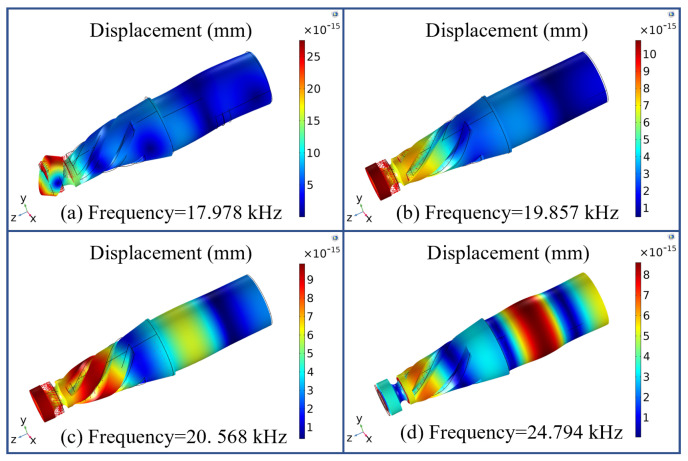
Vibration modes at different frequencies.

The reference value of the characteristic–frequency search was set to 20 kHz. The vibration modes of the four representative characteristic frequencies are shown in Figure 3. According to the experimental requirements, the longitudinal–torsional vibration mode must be output at the end face of the tool. Therefore, the resonance frequency of 19.857 kHz shown in Figure 3b corresponds to the required vibration mode for the experiment, with a 0.7% error compared to the theoretically designed frequency of 20 kHz.

**Figure 4:**
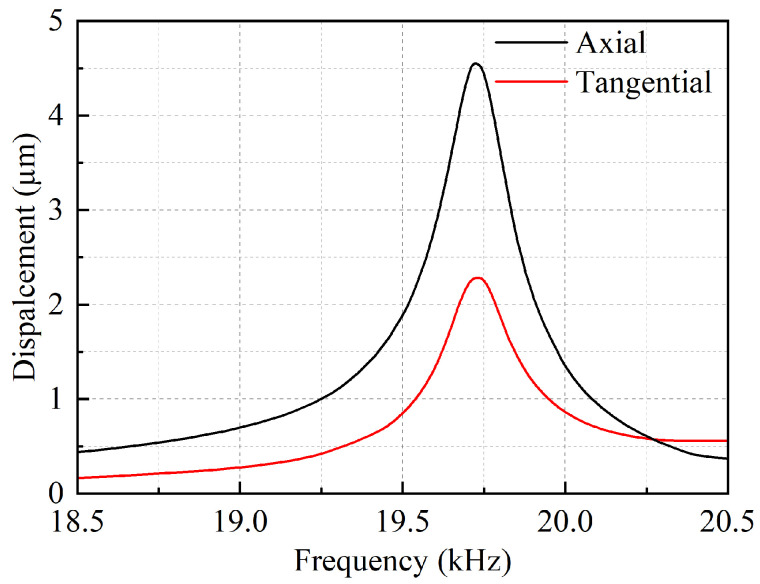
Amplitude simulation results of working end face.

When the system’s excitation voltage is 300 V, the simulation results show the longitudinal–torsional vibration amplitude of the tool end face is shown in Figure 4. The longitudinal vibration amplitude is 4.6 µm and the torsional vibration amplitude is 2.3 µm.

### 2.3. Performance Test of the Vibration System

**Figure 5:**
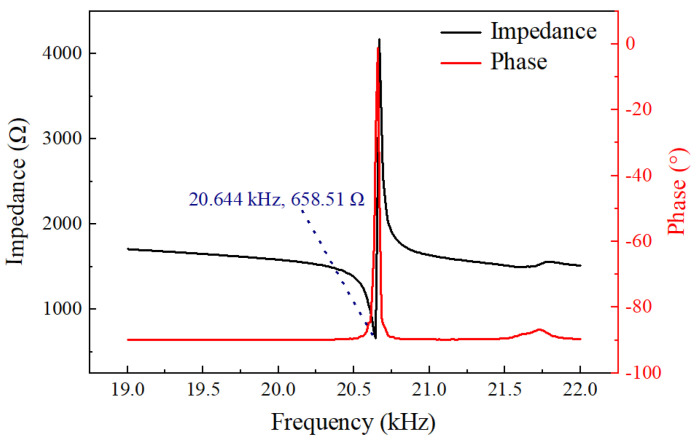
Impedance test results of ultrasonic longitudinal–torsional vibration system.

The impedance characteristics of the prototype were tested using a precision impedance analyzer (6630, MICROTEST, New Taipei, China) and the test results are shown in Figure 5. When the frequency was 20.644 kHz, the impedance of the system reached a minimum value of 658.51 Ω. The 3.9 % deviation between experimental 20.644 kHz and simulated 19.857 kHz resonance frequencies primarily stems from practical machining tolerances and boundary condition differences between simulation ideal fixed constraints and experiment bolt preload effects.

**Figure 6:**
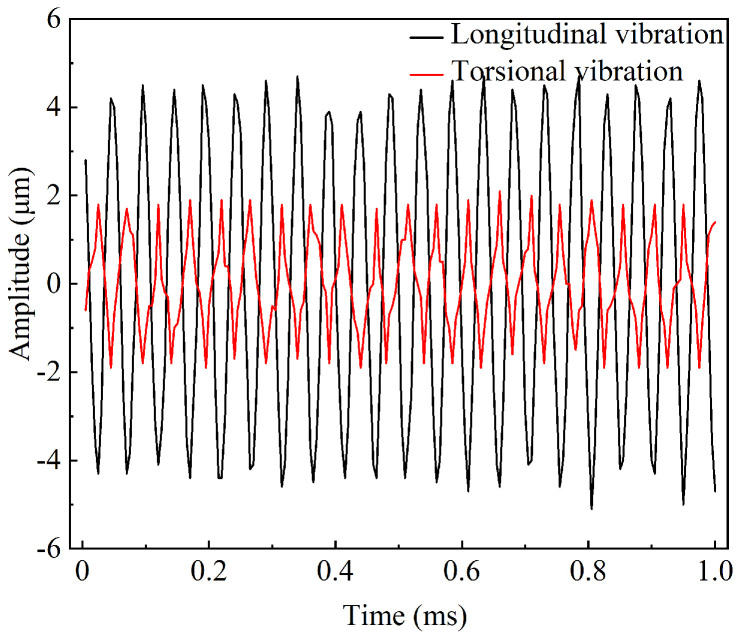
Amplitude test results of working end face.

**Figure 7:**
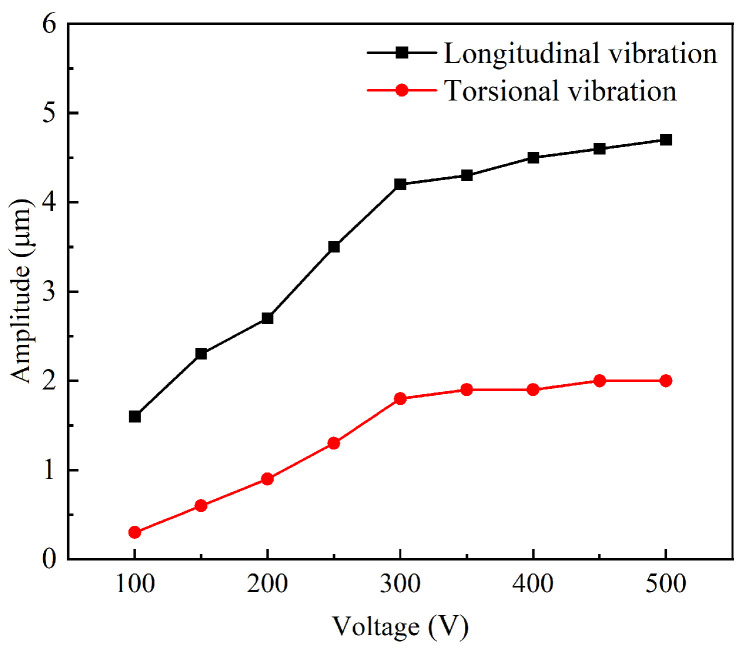
The variation of longitudinal–torsional amplitude with voltage.

The energy transfer between the driving force and the mechanical system is most efficient at the resonance frequency, leading to maximum vibrational amplitude. Ultrasonic machining results in the highest energy transmission efficiency to the workpiece. The minimum impedance at resonance indicates that the system’s resistance to the external driving force is at its lowest, allowing for the most effective conversion of electrical energy into mechanical vibration. Laser displacement sensor (LK-H020, KEYENCE, Osaka, Japan) was used to measure the longitudinal torsional vibration output at the working end face of the ultrasonic longitudinal torsional vibration system under actual operating conditions. The excitation voltage was set to 300 V during the test; the amplitude test results are shown in Figure 6, with the longitudinal amplitude being 4.2 µm and the torsional amplitude being 1.8 µm. The longitudinal–torsional amplitude at the working surface increases with the rise in voltage, as shown in Figure 7. The relationship between the output amplitude and the voltage is not linear. When the voltage was gradually increased in increments of 50 V, the rate of increase of the amplitude was significantly lower than that at 300 V. The piezoelectric and elastic properties of the piezoelectric body are significantly constrained by nonlinear responses under high electric fields. As the voltage further increases, the piezoelectric material of the transducer gradually exhibits nonlinear characteristics, leading to distortion in the frequency response curves of various properties. Consequently, the electro–acoustic conversion efficiency decreases, resulting in a reduced amplitude growth [31,32]. Therefore, to ensure amplitude stability and higher processing efficiency, 300 V was chosen for the study. After comparing the simulation results and considering the effects of bolt preload and prototype errors during processing, the working performance of the ultrasonic vibration system is basically as expected.

### 2.4. Experimental Apparatus and Procedures

**Figure 8:**
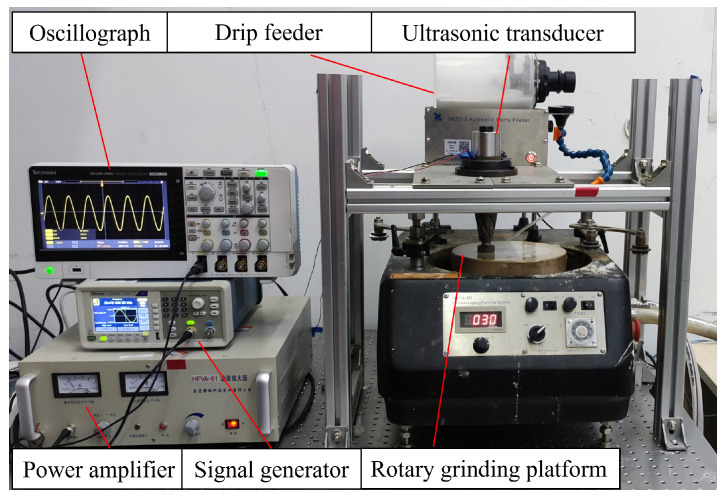
Schematic diagram of ultrasonic longitudinal–torsional grinding.

Figure 8 shows a diagram of the sapphire ultrasonic longitudinal–torsional grinding experimental platform. The experimental platform mainly comprised a signal generator, power amplifier, oscilloscope, ultrasonic longitudinal–torsional vibration system, rotary grinding platform (UNIPOL-802, Shenyang Kejing, Shenyang, China), and drip feeder. The sapphire workpiece sticks to the end face of the tool at a distance from the grinding disk; the properties of sapphire and experimental parameters are presented in Table 2.

In the ultrasonic longitudinal–torsional grinding experiment, the voltage is set to 300 V (peak–to–peak value), drive frequency of the signal generator is set to 20.644 kHz, and the experimental time is 30 min. The control variable method was used to study the influence of clearance, rotational speed, and abrasive particle size on the experimental results. Table 2 lists the process parameters of the sapphire ultrasonic longitudinal–torsional composite grinding experiment, including workpiece characteristics and processing conditions. The drip feeder supplements the grinding fluid consumed in the grinding process, and the supplement speed of the grinding fluid can be adjusted according to the rotational speed in the experiment.

## 3. Results and Discussion

### 3.1. Surface Roughness Reduction and Material Removal Rate

**Figure 9:**
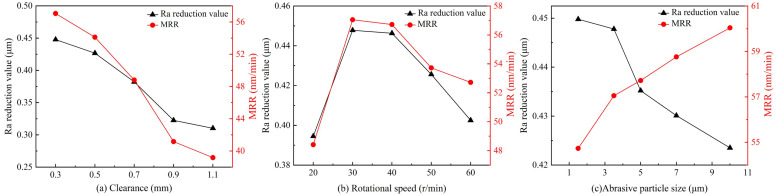
The influencing factors of surface roughness reduction and material removal rate: (**a**) clearance, (**b**) rotation speed, and (**c**) abrasive particle size.

As shown in Figure 9a, the reduction in surface roughness and the material removal rate decrease with increasing processing clearance. When the clearance is set to 0.3 mm, the material removal rate reaches 57.05 nm/min. As the clearance increases, the transmission efficiency of ultrasonic energy through the grinding fluid gradually decreases. This leads to greater attenuation of the abrasive particles’ kinetic energy, weakening their impact on the workpiece and reducing the material removal rate. Furthermore, with a larger clearance, the likelihood of contact between the abrasive particles and the workpiece surface decreases, which further diminishes the effectiveness of reducing surface roughness. As shown in Figure 9b, when the rotation speed is below 30 r/min, the reduction in surface roughness and the material removal rate significantly improve with increased speed. However, once the rotation speed exceeds 30 r/min, the centrifugal force exerted by the grinding fluid becomes greater, causing the abrasives to be expelled from the surface of the grinding disc. The amount of grinding fluid supplied cannot compensate for this loss of abrasives, leading to a reduction in the effectiveness of ultrasonic grinding and, as a result, a decrease in the improvement of surface roughness and material removal rate. As shown in Figure 9c, increasing the size of abrasive particles decreases surface roughness reduction while increasing the material removal rate. The material removal rate reaches its peak at 60.04 nm/min when the abrasive particle size is 10 µm. Larger abrasive particles have a stronger cutting ability, enabling them to remove more significant volumes of material, thereby increasing the material removal rate. However, using larger particles tends to create deeper and more uneven craters on the workpiece surface, reducing the improvement in surface roughness. A comparison of the different experimental groups reveals that the optimal results are achieved with a clearance of 0.3 mm, a rotational speed of 30 r/min, and an abrasive particle size of 3.5 µm.

### 3.2. Surface Morphology of Sapphire

**Figure 10:**
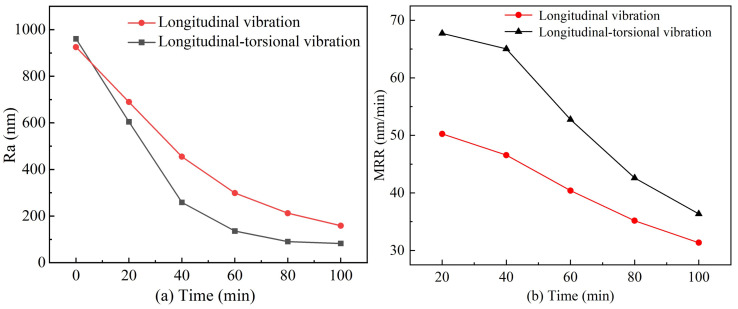
The results of surface roughness and material removal change of sapphire workpiece. (**a**) Surface roughness. (**b**) Material removal rate.

**Figure 11:**
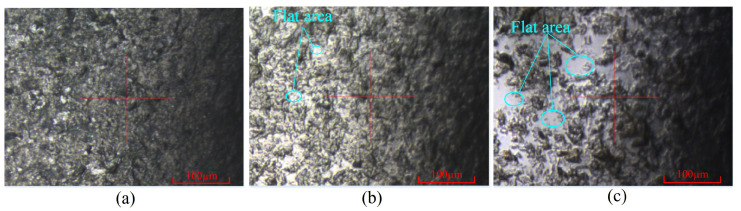
Two-dimensional surface morphology formed under different ultrasonic vibrations. (**a**) Original surface. (**b**) Ultrasonic longitudinal vibration. (**c**) Ultrasonic longitudinal–torsional vibration.

**Figure 12:**
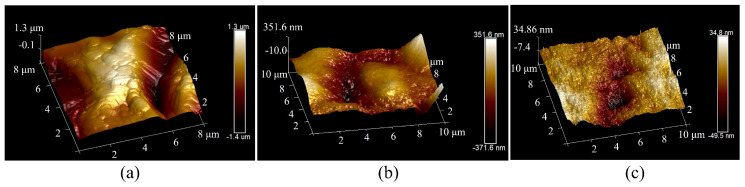
Three-dimensional surface morphology formed under different ultrasonic vibrations. (**a**) Original surface. (**b**) Ultrasonic longitudinal vibration. (**c**) Ultrasonic longitudinal–torsional vibration.

Improving the surface morphology of sapphire wafers is important for ultrasonic longitudinal–torsional grinding experiments. The optimal working conditions for experimenting were determined by a previous study on the influencing factors of the experiment as a clearance of 0.3 mm, a rotational speed of 30 r/min, and abrasive particles of 3.5 µm. A comparative experiment was conducted under identical conditions using ultrasonic longitudinal vibration and a longitudinal–torsional drive. To ensure a fair comparison, the amplitude of the ultrasonic longitudinal vibration system was maintained at the same level as that of the ultrasonic longitudinal–torsional vibration system by adjusting the excitation voltage. The workpiece material and processing parameters were kept consistent. The experiment lasted for 100 min, with the surface roughness of the workpiece measured at 20-min intervals. The results, showing the surface roughness and material removal rate of the sapphire workpiece as functions of experimental time, are presented in Figure 10. Additionally, the two-dimensional and three-dimensional surface morphologies of the workpiece are illustrated in Figure 11 and Figure 12, respectively.

As shown in Figure 10a, after 100 min of the experiment, the surface roughness of the sapphire subjected to ultrasonic longitudinal vibration is reduced from 925.1 nm to 158.8 nm. Another study shows that optical devices with a surface roughness Sa of 0.2 to 0.4 µm can meet the requirements of ultraprecision machining [33]. In current ultrasonic vibration machining systems, ultrasonic–vibration–assisted grinding can reduce the surface roughness of workpieces to a range of 240–680 nm. The ultrasonic longitudinal vibration results align with existing literature on ultrasonic–vibration–assisted grinding [23,34,35]. The experimental results of ultrasonic longitudinal vibration in this study are consistent with the existing ultrasonic vibration grinding system. In contrast, the surface roughness of the sapphire exposed to ultrasonic longitudinal–torsional vibration is reduced from 960.6 nm to 82.6 nm, which is 48% lower than that achieved with ultrasonic longitudinal vibration. The reduction in surface roughness occurs due to ultrasonic longitudinal–torsional vibration, which causes the abrasive particles to move vertically and rotate horizontally. This composite motion increases the contact area and energy transfer path during abrasive grinding so that the tangential kinetic energy of the free abrasive increases so that the surface of the workpiece can be cut more evenly. The change in material removal rate is shown in Figure 10b. As the grinding time increases, the surface roughness of the workpiece gradually decreases, resulting in a smoother surface. The smoother the surface, the lower the material removal ability of the abrasive impact on the surface of the workpiece per unit time. The smoother surface has a smaller cutting depth and fewer rough peaks, which reduces the amount of abrasive cutting, thereby reducing the efficiency of material removal. Comparing the experimental results of longitudinal vibration grinding and longitudinal–torsional vibration grinding, it can be observed that the material removal rate of longitudinal–torsional vibration grinding is consistently higher than that of longitudinal vibration grinding across the entire grinding duration. This difference arises because longitudinal–torsional vibration introduces an additional torsional motion component, which enhances the relative motion between the abrasive grains and the workpiece surface, improving the cutting efficiency. The torsional vibration component increases the abrasive grain trajectory complexity, leading to a larger effective cutting area and a more uniform distribution of abrasive impacts. Consequently, the material removal rate remains higher compared to longitudinal vibration grinding.

As shown in Figure 11, the surface morphology of the sapphire workpiece was characterized using Atomic Force Microscopy (Dimension Icon, BRUKER, Billerica, MA, USA), which provided high–resolution imaging to evaluate the post–grinding surface quality. In contrast, these flat regions were further expanded following ultrasonic longitudinal–torsional grinding, resulting in a more uniform surface flatness. As shown in Figure 12, the surface flatness of the workpiece improved to 723.2 nm after the ultrasonic longitudinal vibration process. The surface flatness was further enhanced to 84.3 nm with ultrasonic longitudinal–torsional grinding, representing an 88.3% improvement over the longitudinal vibration method. The significant improvement in the surface quality is attributed to the combined effect of longitudinal and torsional vibration.

## 4. Conclusions

This paper introduces a precision grinding method for sapphire processing using two-dimensional ultrasonic longitudinal–torsional vibration, demonstrating favorable results. The following conclusions are drawn. The resonant frequency of the ultrasonic longitudinal–torsional horn is 20.644 kHz, with an excitation voltage of 300 V (peak–to–peak). The longitudinal amplitude output by the tool is 4.2 µm and the torsional amplitude is 1.8 µm. The abrasive particles move in the vertical direction while rotating in the horizontal plane under the longitudinal–torsional vibration; this composite motion increases the contact area between the abrasive particles and the workpiece surface, making the impact more uniform and efficient. Experimental results of ultrasonic longitudinal–torsional grinding demonstrate that the surface roughness of sapphire workpieces is reduced from 960.6 nm to 82.6 nm, with an improvement in surface flatness to 84.3 nm. Compared with longitudinal ultrasonic vibration, longitudinal–torsional grinding reduced the surface roughness of sapphire workpieces by 48% and improved the surface flatness by 88.3%, demonstrating the superior synergistic effects of composite vibration modes in minimizing surface defects and enhancing machining precision.

## Figures and Tables

**Table 1:** Materials for components of the ultrasonic vibration system.

Component	Material	Diameter (mm)	Length/Thickness (mm)
Ceramic Ring	PZT-8	50	41
Rear Cover	Stainless Steel	50 (OD) 17 (ID)	6.5
Front Cover	Aluminum Alloy	50	45
Horn	45 Steel	55	85 (Conical)
		25	5 (Cylindrical)
		30	10 (Tool)

**Table 2:** Experimental Parameters.

Experimental Parameters	Constant	Variable
Workpiece material	Sapphire	
Dimension (mm)	25×25×0.48	
Particles material	SiC	
Density (kg/m^3^)	3950	
Initial surface roughness (μm)	0.9–1	
Clearance (mm)	30 r/min, 3.5 μm	0.3, 0.5, 0.7, 0.9, 1.1
Rotational speed (r/min)	3 mm, 3.5 μm	20, 30, 40, 50, 60
Particles size (μm)	3 mm, 30 r/min	1.5, 3.5, 5, 7, 10

## Data Availability

Data are contained within the article.

## References

[1] Liu X., Zhang Q., Chen M., Liu Y., Zhu J., Yang J., Wang F., Tang Y., Zhao X. (2023). Multiphysics modeling and analysis of Sc-doped AlN thin film based piezoelectric micromachined ultrasonic transducer by finite element method. Micromachines.

[2] Tong Z., Wu Z., Gu Y.A., Lou L. (2024). Effect of humid environment on electromechanical performance of piezoelectric micromachined ultrasonic transducers (PMUTs). Sens. Actuators A Phys..

[3] Wang D.-A., Chuang W.-Y., Hsu K., Pham H.-T. (2011). Design of a Bézier-profile horn for high displacement amplification. Ultrasonics.

[4] Yang H., Ji M., Xiu X., Lv H., Gu A., Zhang S. (2022). AlScN film based piezoelectric micromechanical ultrasonic transducer for an extended long-range detection. Micromachines.

[5] Munir M.M., Mughal K.H., Qureshi M.A.M., Qaiser A.A., Khalid F.A. (2024). Design of Novel Longitudinally–Torsionally Coupled Ultrasonic Bezier Horns for Machining Advanced Hard and Brittle Materials. J. Vib. Eng. Technol..

[6] Satpute V., Huo D., Hedley J., Elgendy M. (2023). Design of a novel 2D ultrasonic transducer for 2D high-frequency vibration-assisted micro-machining. Int. J. Adv. Manuf. Technol..

[7] Baraya M., Yan J., Hossam M. (2024). Improving and Predicting the Surface Roughness and the Machining Accuracy in Ultrasonic Vibration-Assisted Milling. J. Vib. Eng. Technol..

[8] Choi Y.-J., Park K.-H., Hong Y.-H., Kim K.-T., Lee S.-W., Choi H.-Z. (2013). Effect of ultrasonic vibration in grinding; horn design and experiment. Int. J. Precis. Eng. Manuf..

[9] Chen F., Bie W., Wang X., Zhao B. (2022). Longitudinal-torsional coupled rotary ultrasonic machining of ZrO_2_ ceramics: An experimental study. Ceram. Int..

[10] Yang Z., Zhu L., Zhang G., Ni C., Lin B. (2020). Review of ultrasonic vibration-assisted machining in advanced materials. Int. J. Mach. Tools Manuf..

[11] Ye Z., Wen X., Wan W., Liu F., Bai W., Xu C., Chen H., Gong P., Han G. (2023). Precision grinding technology of silicon carbide (SiC) ceramics by longitudinal torsional ultrasonic vibrations. Materials.

[12] An D., Huang Y., Li J., Huang W. (2024). Design and Characteristics Study of Longitudinal-Torsional Piezoelectric Ultrasonic Transducers. Int. J. Precis. Eng. Manuf..

[13] Wu C., Chen S., Cheng K., Ding H., Xiao C. (2019). Innovative Design and Analysis of a Longitudinal-Torsional Transducer with the Shared Node Plane Applied for Ultrasonic Assisted Milling. J. Mech. Eng. Sci..

[14] Huang S., Li X., Zhao Y., Sun Q., Huang H. (2021). A novel lapping process for single-crystal sapphire using hybrid nanoparticle suspensions. Int. J. Mech. Sci..

[15] Wang S., Tie G., Shi F., Tian Y., Yang X. (2024). Effect of different grinding strategies on subsequent polishing processes of sapphire. J. Manuf. Process..

[16] Cheng Z., Qin S., Fang Z. (2022). Numerical modeling and experimental study on the material removal process using ultrasonic vibration-assisted abrasive water jet. Front. Mater..

[17] Sharma A.K., Venkatesh G., Rajesha S., Kumar P. (2015). Experimental investigations into ultrasonic-assisted abrasive flow machining (UAAFM) process. Int. J. Adv. Manuf. Technol..

[18] Zarepour H., Yeo S.H. (2012). Single abrasive particle impingements as a benchmark to determine material removal modes in micro ultrasonic machining. Wear.

[19] Qu S., Yu T., Meng F., Zhang C., Zhang X., Ma Z., Wang Z., Yu T., Zhao J. (2023). Material removal rate prediction and surface quality study for ultrasonic vibration polishing of monocrystalline silicon. Int. J. Adv. Manuf. Technol..

[20] Liu Q., Li Q., Chen Z., Li Y., Zhou X., Wang R., Xu P. (2022). Study on abrasive belt grinding process assisted by ultrasonic elliptic vibration. Int. J. Adv. Manuf. Technol..

[21] Wu B., Zhao B., Ding W., Su H. (2021). Investigation of the wear characteristics of microcrystal alumina abrasive wheels during the ultrasonic vibration-assisted grinding of PTMCs. Wear.

[22] Agarwal S. (2015). On the mechanism and mechanics of material removal in ultrasonic machining. Int. J. Mach. Tools Manuf..

[23] Xiang D., Li B., Zhao C., Lei X., Peng P., Yuan Z., Gao G., Jiao F., Zhao B. (2024). Mechanism and experimental evaluation on surface morphology of GCr15SiMn with ultrasonic vibration grinding. Int. J. Adv. Manuf. Technol..

[24] An D., Xian J., Zhang Y., Cheng G., Huang Y., Liang Z., Huang W. (2024). Piezoelectric Ultrasonic Local Resonant Ultra-Precision Grinding for Hard-Brittle Materials. Micromachines.

[25] Xu H., Yin Z., Miao Q., Dai C., Cheng J., Li H., Liang Z., Li Z. (2024). Longitudinal-torsional compound ultrasonic vibration end grinding sapphire: A study on surface topography and roughness. Mater. Sci. Semicond. Process..

[26] Zarepour H., Yeo S.H. (2012). Predictive modeling of material removal modes in micro ultrasonic machining. Int. J. Mach. Tools Manuf..

[27] Ichida Y., Sato R., Morimoto Y., Kobayashi K. (2005). Material removal mechanisms in non-contact ultrasonic abrasive machining. Wear.

[28] Pandey H., Apurva A., Dixit P. (2024). Investigations into velocity decay, initial tool-workpiece gap, and material removal behaviour in ultrasonic micromachining. J. Manuf. Process..

[29] Zhao B., Bie W., Wang X., Chen F., Chang B. (2019). Design and experimental investigation on longitudinal-torsional composite horn considering the incident angle of ultrasonic wave. Int. J. Adv. Manuf. Technol..

[30] Al-Budairi H., Lucas M., Harkness P. (2013). A design approach for longitudinal–torsional ultrasonic transducers. Sens. Actuators A Phys..

[31] Liu Y., Ozaki R., Morita T. (2015). Investigation of Nonlinearity in Piezoelectric Transducers. Sens. Actuators A Phys..

[32] Li Z., Tang L., Yang W., Zhao R., Liu K., Mace B. (2021). Transient Response of a Nonlinear Energy Sink-Based Piezoelectric Vibration Energy Harvester Coupled to a Synchronized Charge Extraction Interface. Nano Energy.

[33] Xu J., He Q., Zhang X., Yi X., Yu Y., Li Y., Zhang L., Gu R., Zhang F. (2024). Investigation into the Role of Si and SiC Phases in RB-SiC Ceramics Surface Modified Ultra-Precision Grinding. Mater. Sci. Semicond. Process..

[34] Chen Y., Su H., Qian N., He J., Gu J., Xu J., Ding K. (2021). Ultrasonic Vibration-Assisted Grinding of Silicon Carbide Ceramics Based on Actual Amplitude Measurement: Grinding Force and Surface Quality. Ceram. Int..

[35] Chen Y., Hu Z., Yu Y., Lai Z., Zhu J., Xu X., Peng Q. (2022). Processing and Machining Mechanism of Ultrasonic Vibration-Assisted Grinding on Sapphire. Mater. Sci. Semicond. Process..

